# Inhibition of MSK1 Promotes Inflammation and Apoptosis and Inhibits Functional Recovery After Spinal Cord Injury

**DOI:** 10.1007/s12031-019-01298-9

**Published:** 2019-03-27

**Authors:** Ze-Xiang Zhong, Si-Si Feng, Shao-Ze Chen, Zhen-Ming Chen, Xuan-Wei Chen

**Affiliations:** 1 Department of Spine Surgery, The First Affiliated Hospital of Fujian Medical University, Fuzhou, China; 2 Department of Pathology and Institution of Oncology, The School of Basic Medical Sciences,Fujian Medical University, Fuzhou, China

**Keywords:** MSK1, Spinal cord injury, Inflammation, Apoptosis, Functional recovery

## Abstract

Mitogen- and stress-activated kinase (MSK) 1 is a nuclear serine/threonine kinase. In the central nervous system, it plays an important role in regulating cell proliferation and neuronal survival; it is also involved in astrocyte inflammation and the inhibition of inflammatory cytokine production. However, its specific role in spinal cord injury is not clear. Here, we aimed to elucidate this role using an in vivo animal model. In this study, we found that MSK1 is gradually decreased, starting 1 day after spinal cord injury and to its lowest level 3 days post-injury, after which it gradually increased. To further investigate the possible function of MSK1 in spinal cord injury, we interfered with its expression by utilizing a small interfering RNA (siRNA)-encoding lentivirus, which was injected into the injured spinal cord to inhibit local expression. After MSK1 inhibition, we found that the expression of tumor necrosis factor (TNF)-α, interleukin (IL)-6, and IL-1β were increased. Moreover, the expression of IL-10 was decreased. In addition, neuronal apoptotic cells were increased significantly and expression of the apoptosis-related protein caspase-3 was also increased. Ultrastructural analysis of nerve cells also revealed typical neuronal apoptosis and severe neuronal damage. Finally, we found that hindlimb motor function decreased significantly with MSK1 knockdown. Therefore, our findings suggest that the inhibition of this protein promotes inflammatory responses and apoptosis and suppresses functional recovery after spinal cord injury. MSK1 might thus play an important role in repair after spinal cord injury by regulating inflammation and apoptosis.

**Electronic supplementary material:**

The online version of this article (10.1007/s12031-019-01298-9) contains supplementary material, which is available to authorized users.

## Introduction

Spinal cord injury (SCI) is the most serious complication of spinal injury and often leads to severe dysfunction of the lower extremities of the injured segment. Despite the clinical application of drug intervention, surgical treatment, and modern rehabilitation training, no ideal curative effect has been achieved (Rouanet et al. 2017). The reason for this is the lack of effective interventions for pathological changes that occur after SCI, which include primary and secondary injury (Saghazadeh and Rezaei 2017). Primary injury occurs immediately after the initial injury and is irreversible. The pathological process includes demyelination of the spinal cord and neuronal necrosis (Wang et al. 2017). Secondary injury is the main cause of aggravated neurological dysfunction; in contrast, it is reversible and can be regulated. The pathological process for this includes microcirculatory disturbance, excitatory amino acid release, inflammatory reactions, apoptosis, oxygen free radical damage, lipid peroxidation, and astrogliosis (Ueno and Yamashita 2008). Secondary injury to SCI involves multiple pathways such as the mitogen-activated protein kinase (MAPK) signaling, which induces a cascade that amplifies the inflammatory response, ultimately leading to neuronal necrosis (Qu et al. 2012; Deak et al. 1998). Mitogen- and stress-activated kinases (MSKs) 1 and 2 are nuclear serine/threonine protein kinases that are downstream of the extracellular regulated protein kinases (ERK)1/2 or p38 MAPK pathways (Arthur 2008). MSK1 is activated by ERK and typically mediates the biological effects of growth factors, peptide hormones, neurotransmitters, and cytokines (Cheng et al. 2013). MSK1 is also activated by p38 and typically mediates responses to cellular inflammatory stimuli and stress such as UV irradiation and oxidative stress (Zhu et al. 2014). In addition, Toll-like receptors (TLRs) recognize pathogenic microorganisms such as bacterial lipopolysaccharide (LPS) and also activate MSK1 (Ananieva et al. 2008). This kinase directly targets cAMP-response element binding protein (CREB) (Simon et al. 2004; Kaiser et al. 2007) and nuclear factor (NF)-κB p65 (Vermeulen et al. 2003), two important transcription factors involved in the regulation of inflammatory genes, thereby enhancing their transcriptional activity (Vermeulen et al. 2009). MSK1 also phosphorylates histone H3 (Kim et al. 2008) and transcription factor 1 (ATF1) (Wiggin et al. 2002), which have a major role in the regulation of specific subsets of immediate early genes (Arthur 2008). MSK1 is widely involved in immune and inflammatory responses (Reyskens and Arthur 2016), plays an important role in regulating cell proliferation and transformation, and regulates synaptic plasticity, neuronal survival, neuronal maturation, and developmental plasticity (Arthur 2008). Although MSK1 is expressed in a variety of tissues, its level is high in immune and neuronal cells. In the CNS (central nervous system), MSK1 is involved in astrocyte inflammation and inhibits the production of inflammatory cytokines (Gong et al. 2013); moreover, it might be involved in the pathophysiology of traumatic brain injury (TBI) (Ning et al. 2013). MSK1 was also suggested to contribute to SAH (subarachnoid hemorrhage)-induced apoptosis (Ning et al. 2017). Further, recent research has demonstrated its expression in the spinal cord (Li et al. 2013), but its specific role in spinal cord injury is still not clear. Therefore, in the present study, we used a recombinant lentiviral vector to inhibit MSK1 expression in the injured spinal cord to explore the possible effects of MSK1 downregulation on the inflammatory response, apoptosis, and motor function after acute spinal cord injury.

## Materials and Methods

### Lentiviral Vector

The MSK1 small interfering RNA (MSK1-siRNA) recombinant lentiviral vectors were purchased from and constructed by Gene Pharma Co., Ltd. (Shanghai, China). Three shRNA sequences that target the rat *MSK1* sequence (GenBank NM_001108048.1) were designed as follows: 5′-GCGTTTCACAGAGCACGAAGT-3′ (KD1), 5′- GGAATGAGCTCAGTAGCTAAA-3′ (KD2), and 5′-GCTCCTTCCATCCTCTTCAAG-3′ (KD3). These lentiviral vectors were generated by cotransfecting 293T cells with four plasmids (vector plasmid, pGag/Pol(gag-pol plasmid), pRev (rev plasmid), and pVSV-G4 (envelope plasmid pseudotyped with glycoprotein of the vesicular stomatitis virus)). The vector plasmids were constructed with double-stranded DNA formed by annealing the Oligo DNA target sequence with the LV3 (pGLV3/H1/GFP+Puro) vector digested with *Eco*RI and *Bam*HI (see the Electronic Supplementary Material for more details on the construction of lentivirus).

After transfection and culture for 72 h, the medium containing lentiviral vectors was obtained and concentrated via ultracentrifugation (4 °C, 20,000 rpm for 2 h). Then, the lentiviral suspensions were transfected into 293T cells at different dilutions. The titer of vectors was determined by measuring the number of fluorescent cells by fluorescence microscopy and combined with the lentiviral dilution ratio. The titers of MSK1 siRNA lentiviral vectors were observed to be 0.9 × 10^9^ TU/mL, which was then diluted to 0.5 × 10^9^ TU/mL. A lentiviral vector that only expresses green fluorescent protein (LV-GFP) was also generated as a negative control. The titer was 0.5 × 10^9^ TU/mL for LV-GFP.

### Animal Model of Spinal Cord Injury

A total of 72 male Sprague–Dawley (SD) rats weighing 200–250 g were purchased from SLAC Laboratory Animal Co., Ltd. (Shanghai, China). All animal studies were approved by the Experimental Animal Ethics Committee of Fujian Medical University in China. All experimental procedures were performed in accordance with the National Institutes of Health guidelines for the Care and Use of Laboratory Animals. All rats were anesthetized with 2% sodium pentobarbital (30 mg/kg, i.p.), and the spinal cord, which was approximately 1.0 cm long, was exposed by removing the T9 and T11 lamina. NYU Impactor-III impactor (W.M. Keck, America) was used to create a rat spinal cord injury model according to Allen’s method (Allen 1911). T10 spinal cords were then impacted by a heavy object (10 g in weight and 2.5 cm in height). The successful injury model was evaluated according to the spasm swing of the tail, spastic jitter of both lower limbs, subdural congestion, and loss of hindlimb motor function.

### Experimental Design

#### Experiment 1

Thirty male SD rats were randomly divided into normal group (*n* = 5) and SCI group (*n* = 25). SCI was induced in rats in the SCI group according to Allen’s method. For the normal group, the rats did not do any treatment. Spinal cord tissue was collected at 1, 3, 5, 7, and 14 days after SCI; each group of five rats was used to detect the expression of MSK1 by western blot assays.

#### Experiment 2

Forty-two male SD rats were randomly divided into the following groups with 14 rats in each group: SCI group (rats were subjected to SCI), LV-GFP group (SCI rats that received a spinal cord injection of negative control lentivirus (0.5 × 10^9^ TU/mL), and LV-MSK1 group (SCI rats that received a spinal cord injection of MSK1-siRNA lentivirus (0.5 × 10^9^ TU/mL). For the LV-GFP and LV-MSK1 groups, rats were subjected to spinal cord injury, and then lentivirus was immediately injected into the injured spinal cord. A total of 6 μL of lentivirus (approximately 3 × 10^6^ TU MSK1-siRNA or LV-GFP) solution was injected at two different locations (3 μL each; 2.0 mm caudal and 2.0 mm rostral from the lesion epicenter) at a depth of 0.8 mm using a 10-μL microinjector according to the local intraspinal injection method (Hu et al. 2016; Tan et al. 2017). To promote lentiviral absorption, the rate of injection was 1 μL/min, and the pipette was left in place for at least 5 min to prevent leakage.

### Cell Culture and Lentiviral Transduction

Well-differentiated PC12 cells induced by nerve growth factor were obtained from the Cell Resource Center of Shanghai Institutes for Biological Sciences, Chinese Academy of Sciences (Shanghai, China). Well-differentiated PC12 cells are widely used in vitro as a model to investigate neuronal damage resulting from various hypoxia-related neurodegenerative disorders and spinal cord injury (Lin et al. 2016; Liu et al. 2016; Pan et al. 2017; Goldshmit et al. 2018; Xu et al. 2018). Well-differentiated PC12 cells were cultured in RPMI-1640 medium containing 10% heat-inactivated fetal bovine serum at 37 °C in a humidified atmosphere with 5% CO_2_. For further experiments, Well-differentiated PC12 cells were seeded into 24-well plates at a density of 5 × 10^4^ cells/mL. Then, the lentivirus solution (MSK1-siRNA and LV-GFP) was added at an MOI of 100. After 72 h of culture in the original medium, the transduction efficiency was observed with a fluorescence microscope. After 96 h, cells were harvested for protein extraction and subsequent western blotting to assess the expression of MSK1. The most efficient recombinant MSK1-siRNA lentivirus was used for subsequent studies.

### Western Blot Analysis

To obtain samples for western blot analysis, 1.0-cm-long spinal cord tissues containing the epicenter were quickly dissected at 7 days post-injury and snap frozen at − 80 °C until use. The samples were cut into small pieces on ice, added to a cocktail of RIPA lysis buffer with the protease inhibitor PMSF, and homogenized with a glass homogenizer until fully lysed. After determining the protein concentration with a BCA protein assay kit (Beyotime, Shanghai, China), equivalent amounts of protein were separated by 10–12% SDS-PAGE. The proteins were transferred to PVDF membranes (Millipore, USA) using a transfer apparatus at 110 V for 1 h. The membrane was blocked with 5% nonfat milk at room temperature for 1 h and incubated with primary antibodies including anti-MSK1 (1:1000, Novus, USA), anti-TNF-α (1:500, Abcam, USA), anti-IL-6 (1:1000, Abcam, USA), anti-IL-10 (1:1000, Abcam, USA), anti-IL-1β (1:500, Abcam, USA), anti-GAPDH (1:1000, CST, USA), and anti-β-actin (1:1000, Santa Cruz, USA) at 4 °C overnight. After washing with TBST, the membranes were incubated with secondary goat anti-rabbit IgG-HRP antibody (1:3000, Servicebio, Hubei, China) for 2 h at room temperature. The supersensitive ECL Chemiluminescence Kit (Beyotime, Shanghai, China) was used to expose images using a gel imaging system. Semi-quantitative analysis was performed using Image J software.

### Immunofluorescence Staining

At 7 days post-injury, rats were anesthetized and transcardially perfused with physiological saline and 4% paraformaldehyde. Spinal cords were rapidly isolated and post-fixed in 4% paraformaldehyde for 8 h at 4 °C, which was followed by immersion in phosphate buffer containing 30% sucrose until the sample sank. The spinal cords were then embedded in OCT solution, and 20-μm coronal spinal cord cryosections were prepared.

For immunofluorescence double staining, we first added an auto-fluorescent quencher to the sections to prevent autofluorescence. Then, the sections were blocked with 10% normal serum blocking solution, which was the same species as the secondary antibody to avoid unspecific staining. The sections were incubated at 4 °C overnight with the following primary antibodies: rabbit anti-MSK1 (1:100, Novus, USA), rabbit anti-caspase-3 (1:100, Abcam, USA), and mouse anti-neuronal nuclei (NeuN) (1:100, Abcam, USA). After washing in PBS, sections were incubated with an appropriate secondary antibody for 50 min at room temperature, including Cy3-conjugated goat anti-rabbit IgG (1:300, Servicebio, Hubei, China) and Alexa Fluor 488-conjugated goat anti-mouse IgG secondary (1:400, Servicebio, Hubei, China) antibodies. The nuclei were then stained with DAPI (Servicebio, Hubei, China). The stained sections and the green auto-fluorescence induced by the lentiviral vector were visualized with a fluorescence microscope (Nikon, Tokyo, Japan).

### TUNEL

TUNEL analysis was performed according to the instructions of the fluorescent TUNEL kit (Roche, Basel, Switzerland). The kit was based on FITC fluorescein labeling and positive apoptotic nuclei appeared green by fluorescence microscopy. The sections were then immersed in cold acetone for 10 min. After washing with PBS (pH 7.4), the sections were incubated with proteinase K working solution at 37 °C for 25 min and Broken membrane working solution at room temperature for 20 min. After being washed with PBS (pH 7.4), The mixture of reagent 1 (TdT) and reagent 2 (dUTP) in a 1:9 ratio was immense the sections for 2 h at 37 °C. The nuclei were stained with DAPI at room temperature for 10 min. Images were examined by a fluorescence microscopy (Nikon, Tokyo, Japan). Positive cell counts and analysis were performed using ImageJ software (1.51, NIH).

### Transmission Electron Microscopy

At 14 days post-injury, rats were anesthetized and transcardially perfused with physiological saline. Spinal cords were rapidly isolated and post-fixed in 2.5% glutaraldehyde for 4 h at 4 °C, and then transferred to 1% osmic acid for 2 h at room temperature. After dehydration in increasing concentrations of acetone, the tissues were embedded. Then, the samples were inserted into an embedding plate and placed into a dry at 37 °C overnight, followed by 60 °C for 48 h. The spinal cords were cut into 60–80-nm slices with an Ultrathin microtome (Leica USA), and the sections were stained with 2% saturated uranyl acetate solution and lead citrate overnight at room temperature. Images were observed and collected using a transmission electron microscope (Hitachi USA).

### Evaluation of Motor Function

The hind limb motor function was assessed using the Basso, Beattie, and Bresnahan (BBB) scores (Basso et al. 2009). The main observation index was the activity and coordination of hind limbs, tail, and posterior trunk. Rats were placed on a wide platform and allowed free exercise for 5 min. All functional scores were obtained at 1, 3, 5, 7, and 14 days after operation by two individuals blinded to treatment. The average of the two scores was the final BBB score, to reduce score error.

### Statistical Analysis

Statistical analysis was performed using SPSS v19.0 software. Data were expressed as the mean ± standard deviation (*x* ± *s*). The mean of each group was compared by one-way ANOVA. The LSD-t test was used for multiple comparisons. The test level was α = 0.05.

## Results

### MSK1 Is Downregulated After SCI

**Fig. 1:**
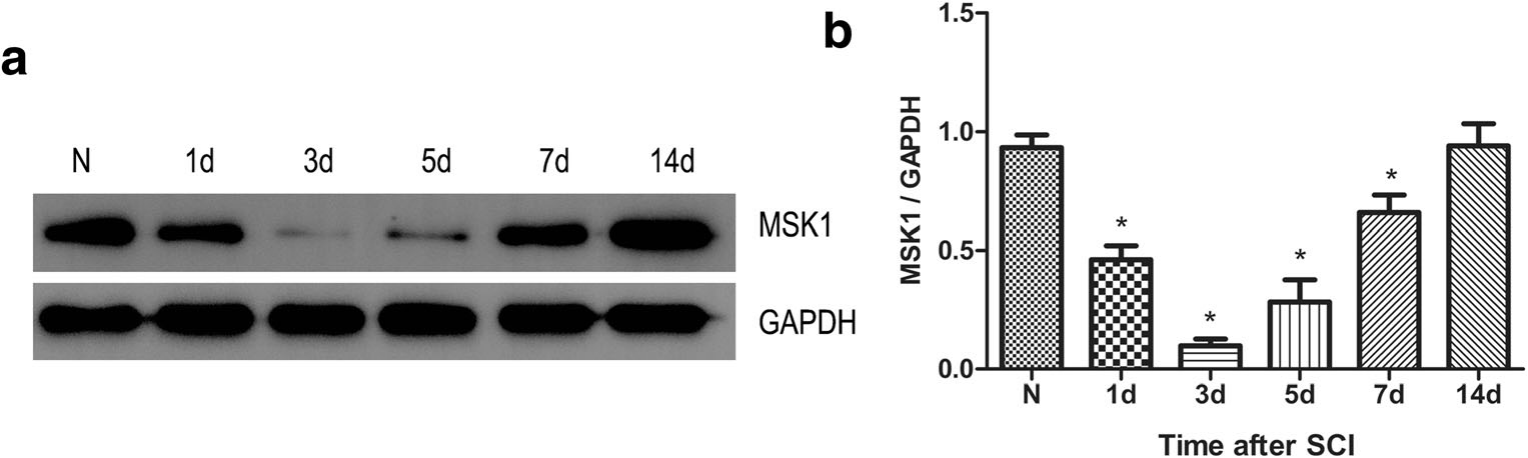
Changes for MSK1 expression at various survival times following SCI. **a** Representative western blot picture showing MSK1 expression after SCI. **b** Quantitative analysis of relative expression of MSK1 protein for each time point. N denotes cortical extracts from normal rats. The data are mean ± SD (*n* = 5, *P < 0.05 vs. normal groups)

We first examined the expression level of MSK1 at different time points after spinal cord injury (Fig. 1). We found that the expression of MSK1 protein was gradually decreased after SCI at 1 day post-injury, to its lowest level at 3 days post-injury. However, after which it gradually increased at 5 and 7 days post-injury, to reach normal levels at 14 days after injury. The expression of MSK1 in the SCI group was significantly lower than that in the normal group at 1, 2, 3, 5, and 7 days after injury (*P* < 0.05).

### The Expression of MSK1 Protein in Well-Differentiated PC12 Cells Infected with Lentivirus

To verify interference efficiency, we infected well-differentiated PC12 cells with MSK1 siRNA recombinant lentivirus. The differentiated PC12 cells exhibit preserved dopaminergic characteristics and spindle-shaped cell morphology similar to neuronal cells. Well-differentiated PC12 cells are widely used in vitro as a model to investigate neuronal damage resulting from various hypoxia-related neurodegenerative disorders.

**Fig. 2:**
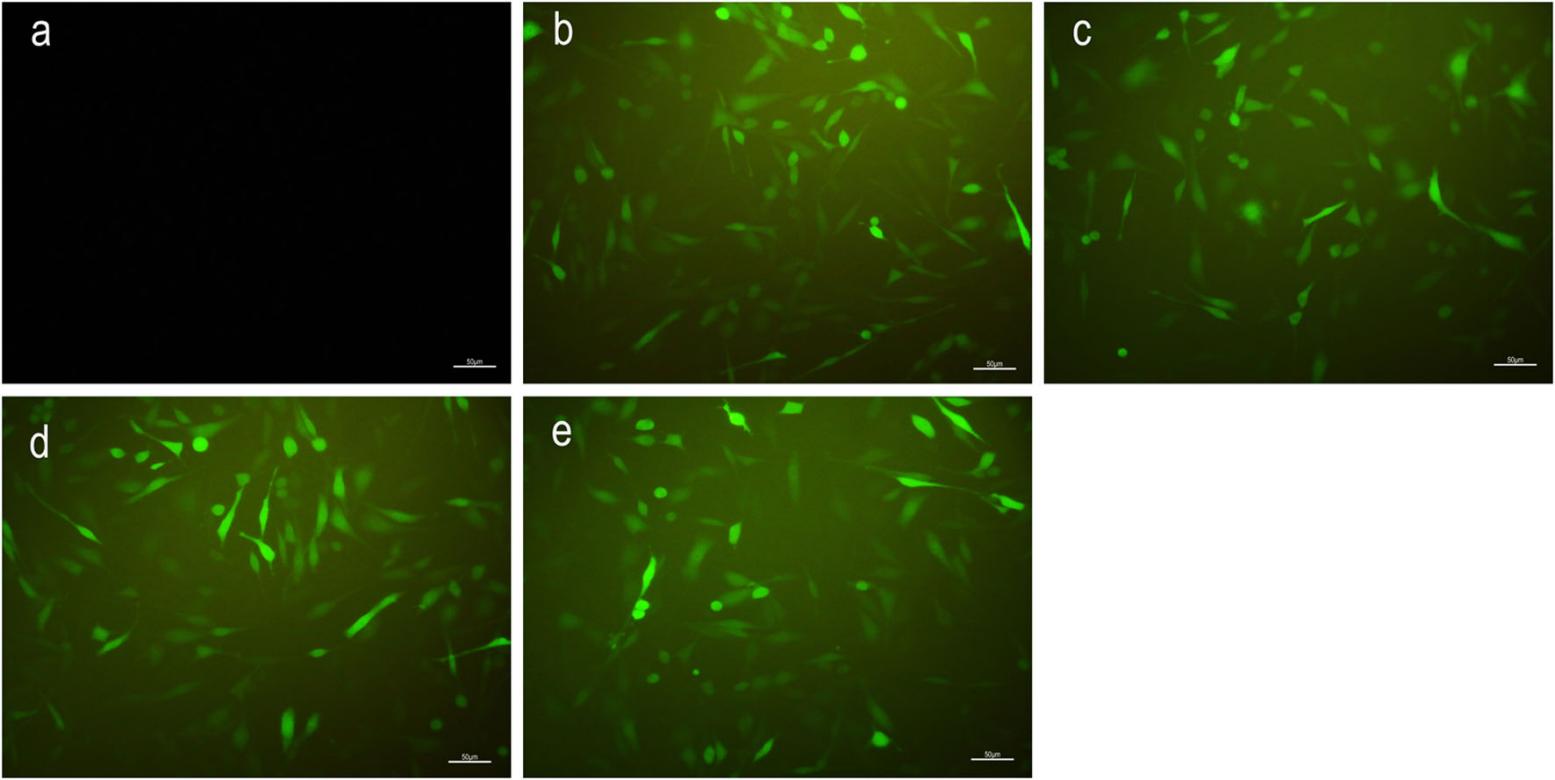
Fluorescence microscopy images showing the expression of green fluorescent protein (GFP) in well-differentiated PC12 cells transfected with recombinant lentivirus vector for 72 ;h. **a** Untreated with lentivirus. **b**–**e** Transfection with LV-GFP, MSK1-siRNA1, MSK1-siRNA2, and MSK1-siRNA3 lentiviral vectors

Well-differentiated PC12 cells were transduced with lentivirus and 72 h later, GFP expression was evaluated using a fluorescence microscope. Results revealed that compared with that in the NC group, a large amount of green fluorescence was observed in the MSK1-siRNA1, MSK1-siRNA2, MSK1-siRNA3, and LV-GFP groups (Fig. 2). Approximately 80% of the cells were infected with the recombinant lentiviral supernatant.

**Fig. 3:**
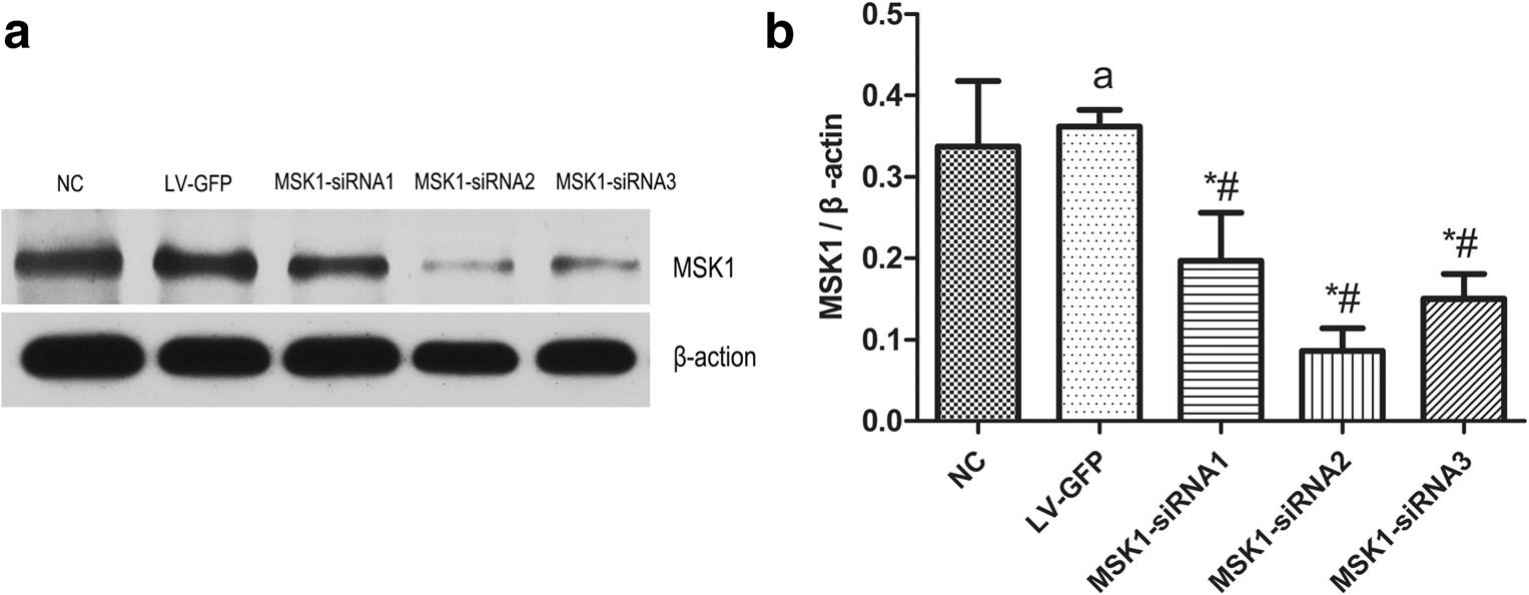
The expression of MSK1 protein in well-differentiated PC12 cells infected with lentivirus after 96 h. **a** Representative western blot picture showing expression of MSK1. **b** Quantitative analysis of relative expression of MSK1 protein. The data are mean ± SD (*n* = 3, *P < 0.05 vs. NC group, ^#^*P* < 0.05 vs. LV-GFP group, ^a^*P* > 0.05, vs. NC group)

Ninety-six hours after well-differentiated PC12 cells were infected with lentivirus, the expression of MSK1 protein was measured by western blotting, as shown in Fig. 3. Expression in the MSK1-siRNA1, MSK1-siRNA2, and MSK1-siRNA3 groups was significantly lower than that in the NC and LV-GFP groups (all *P* < 0.05). There was no significant difference between the NC and LV-GFP groups (*P* > 0.05). This indicated that the constructed MSK1-siRNA lentiviral vector could successfully inhibit the expression of MSK1 in well-differentiated PC12 cells. It is worth noting that the MSK1-siRNA2 lentivirus vector had the highest interference efficiency compared with expression in the LV-GFP group. Therefore, the MSK1-siRNA2 lentivirus vector was selected for follow-up studies.

### Lentiviral Injection into Injured Spinal Cords Inhibits MSK1 Expression

Next, we injected MSK1 siRNA lentivirus suspensions into contused spinal cords, using LV-GFP lentivirus as a control. Fluorescence microscopy was employed to detect auto-fluorescence emitted by GFP to assess the efficacy of lentiviral gene delivery and western blotting was performed to determine the expression of MSK1 protein 7 and 14 days after the injection.

**Fig. 4:**
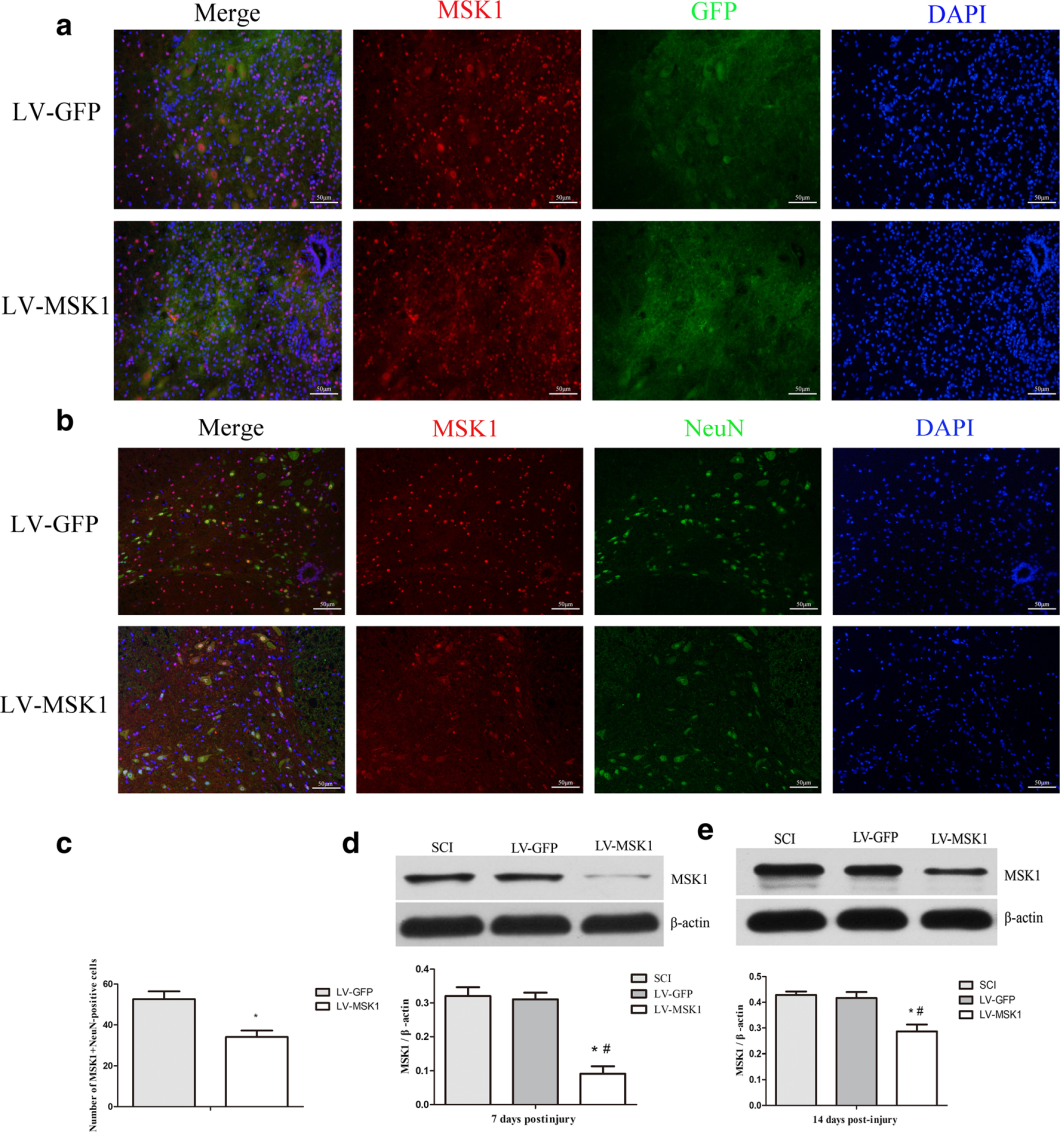
Lentiviral injection into injured spinal cords inhibits MSK1 expression. **a** Representative image of green auto-fluorescent of GFP (green) and immunofluorescence staining of MSK1 (red) in LV-GFP and LV-MSK1 groups. **b** Immunofluorescence double staining of MSK1 (red) sand NeuN (green) in LV-GFP and LV-MSK1 groups. **c** Quantitative analysis of the number of MSK1+NeuN-positive cells in LV-GFP and LV-MSK1 groups. **d**, **e** Western blot and quantitative of relative expression of MSK1 protein in injured spinal cord 7 and 14 days after lentiviral injection. The data are mean ± SD (*n* = 3, *P < 0.05 vs. LV-GFP group, ^#^*P* < 0.05 vs. SCI group)

Fluorescence microscopy showed that spontaneous green fluorescence was observed in both LV-GFP and LV-MSK1 groups (Fig. 4a). Moreover, the green fluorescence conferred by GFP colocalized with the red fluorescence mediated by immunofluorescence staining of MSK1. This indicates that MSK1 was expressed in the spinal cord and that it colocalized with GFP. Immunofluorescence double staining showed that MSK1 colocalized with NeuN (Fig. 4b). Quantitative analysis showed that the number of MSK1+NeuN-positive cells were decrease in the LV-MSK1 group compare with that in LV-GFP group (*P* < 0.05) (Fig. 4c). Additionally, at 7 and 14 days after the injection, MSK1 proteins expression in the LV-MSK1 group were significantly lower than that in the SCI and LV-GFP groups (both *P* < 0.05) (Fig. 4d, e). There was no significant difference in the expression of MSK1 between the SCI and LV-GFP groups (*P* > 0.05). These data provide evidence that lentiviral injection can successfully transduce cells in the spinal cord and inhibit MSK1 expression following SCI.

### Inhibition of MSK1 Promotes Inflammation After SCI

**Fig. 5:**
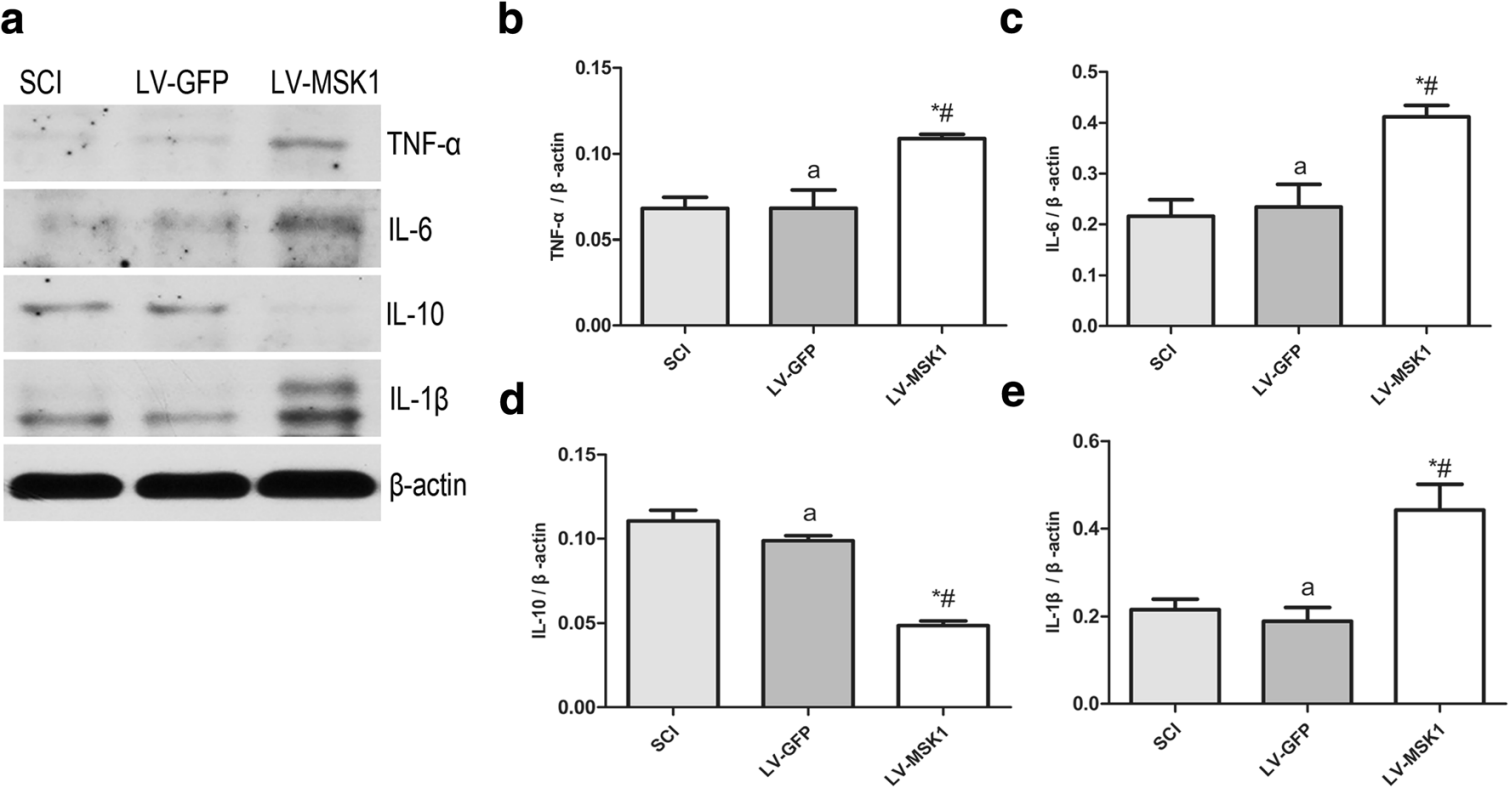
Inhibition of MSK1 promotes inflammation after SCI. **a** Representative western blot picture showing the expression of TNF-α, IL-6, IL-10, and IL-1β in the SCI, LV-GFP, and LV-MSK1 groups at 7 days post-injury. **b**–**e** Quantitative analysis of relative expression of TNF-α, IL-6, IL-10, and IL-1β. The data are mean ± SD (*n* = 3, *P < 0.05 vs. SCI group, ^#^*P* < 0.05 vs. LV-GFP group, ^a^*P* > 0.05, vs. SCI group)

To investigate whether MSK1 inhibition affects inflammation, the expression of TNF-α, IL-6, IL-10, and IL-1β was measured by western blotting. This showed that the expression of TNF-α, IL-6, and IL-1β in the LV-MSK1 group was significantly higher than that in the SCI and LV-GFP groups (all *P* < 0.05; Fig. 5a–d). The expression of IL-10 in the LV-MSK1 group was significantly lower than that in SCI and LV-GFP groups (both *P* < 0.05; Fig. 5e). There was no significant difference between the SCI and LV-GFP groups (*P* > 0.05). These data indicate that inhibition of MSK1 promotes inflammation.

### Inhibition of MSK1 Promotes Apoptosis After SCI

**Fig. 6:**
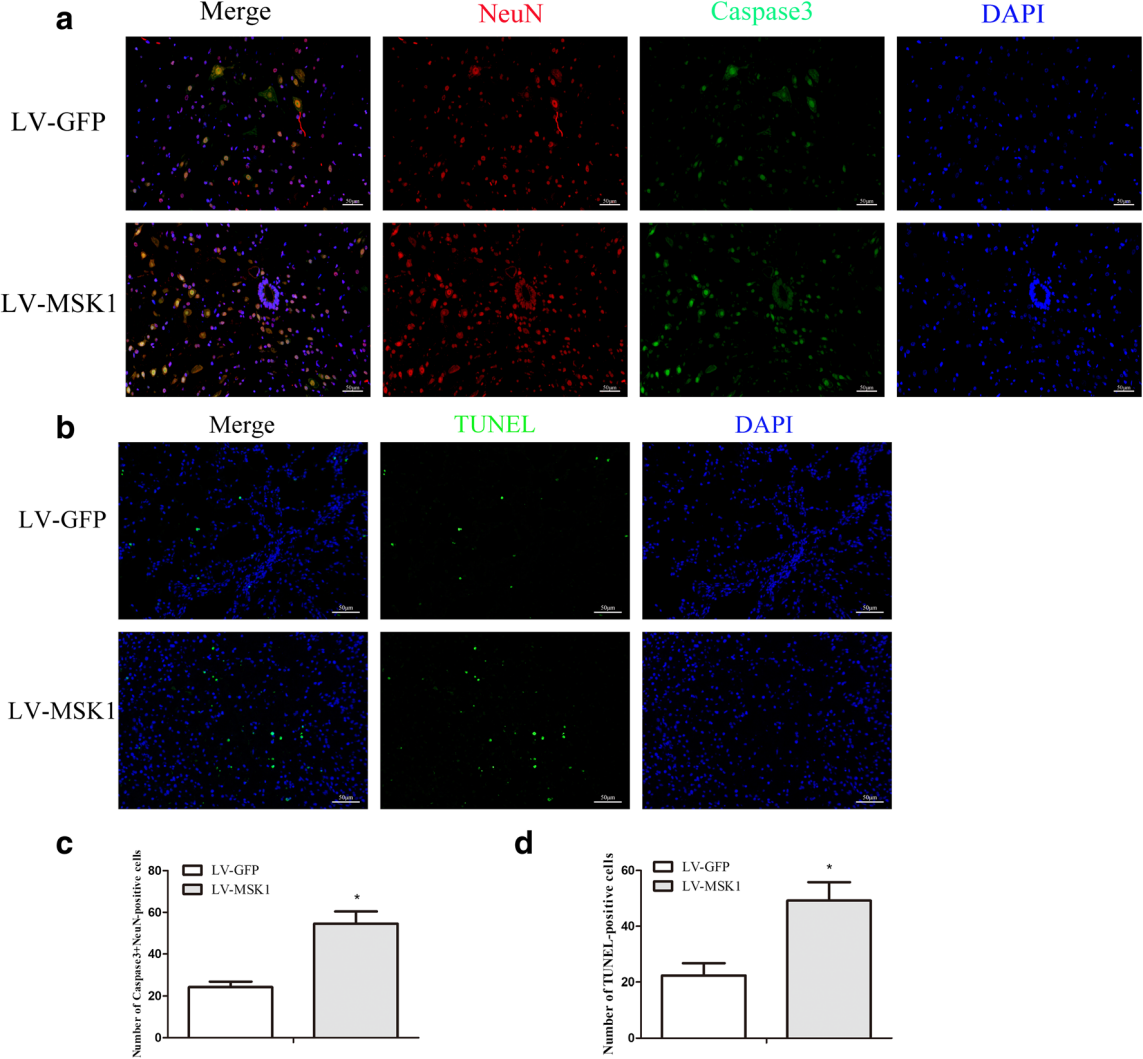
Inhibition of MSK1 promotes apoptosis after SCI. **a** Representative images of immunofluorescence double staining for NeuN (red) and caspase-3 (green) at 7 days post-injury. **b** TUNEL assay analysis of cell apoptosis in LV-GFP and LV-MSK1 group at 7 days post-injury. **c**, **d** Quantitative estimation of the number of NeuN^+^ caspase-3- and TUNEL-positive cells. The data are mean ± SD (*n* = 3, *P < 0.05 vs. LV-GFP group)

**Fig. 7:**
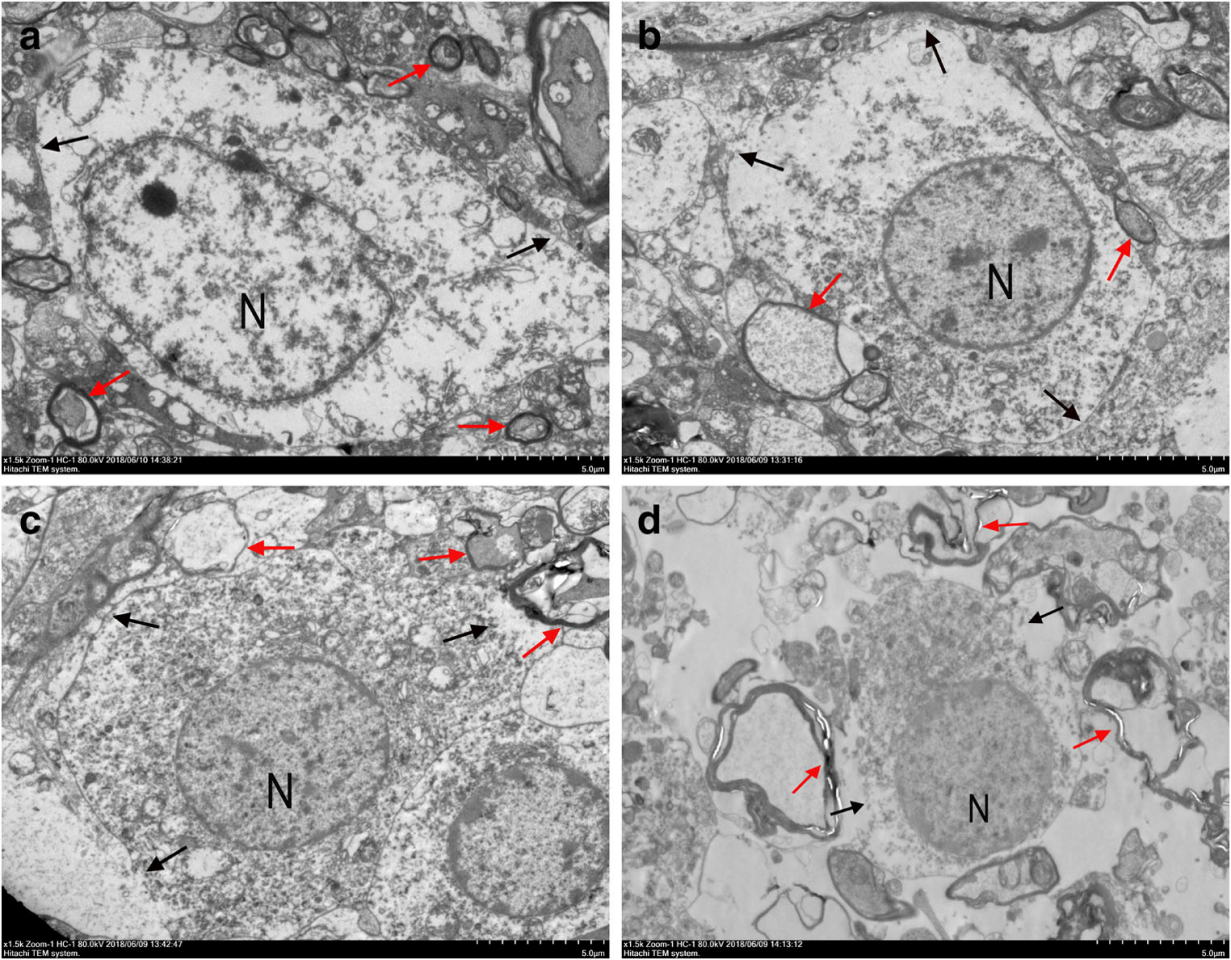
Ultrastructural analysis of spinal neurons by transmission electron microscopy (TEM) at 14 days post-injury. **a** Neurons in normal spinal cord with intact cell and nuclear membranes, uniform nuclear chromatin, regular myelin, and dense laminate. **b**, **c** Neurons in the SCI and LV-GFP group with typical characteristics of apoptosis, such as condensed nuclei, concentrated chromatin, wrinkled nuclear membrane, irregular shape, and thin myelin. **d** Neurons in the LV-MSK1 group with severely damaged cell membranes and structurally disrupted myelin with demyelination (N, nucleus; red arrow, myelin; black arrow, cell membrane. Photomicrographs represent samples taken from three rats in each group)

To investigate whether inhibition MSK1 affects apoptosis, we employed immunofluorescence staining to detect the expression of the apoptosis-related protein caspase-3. TUNEL staining were also performed to detect apoptosis, whereas ultrastructural changes of spinal cord neurons were observed by transmission electron microscopy (TEM). Immunofluorescence double staining showed that the number of NeuN^+^ caspase-3-positive cells in the LV-MSK1 group was significantly higher than that in the LV-GFP group (*P* < 0.05; Fig. 6a, c). In addition, TUNEL staining showed that the number of TUNEL-positive cells in the LV-MSK1 group was evidently increased compared with that in the LV-GFP group (*P* < 0.05; Fig. 6b, d). TEM revealed that in the normal spinal cord (Fig. 7a), neurons exhibited intact structure, as shown by intact cell and nuclear membranes, uniform nuclear chromatin, regular myelin, dense lamellae, and regular mitochondria. In contrast, in the SCI and LV-GFP groups (Fig. 7b, c), neurons exhibited typical characteristics of apoptosis, such as condensed nuclei, concentrated chromatin, wrinkled nuclear membrane, irregular shape, and thin myelin. However, in the LV-MSK1 group (Fig. 7d), neurons were found to have severely damaged cell membranes, swollen mitochondria, and structurally disrupted myelin with demyelination. These results suggest that neurons suffered significant damage and demonstrated that inhibition of MSK1 aggravates apoptosis after SCI.

### Inhibition of MSK1 Reduces Motor Functional Recovery After SCI

**Fig. 8:**
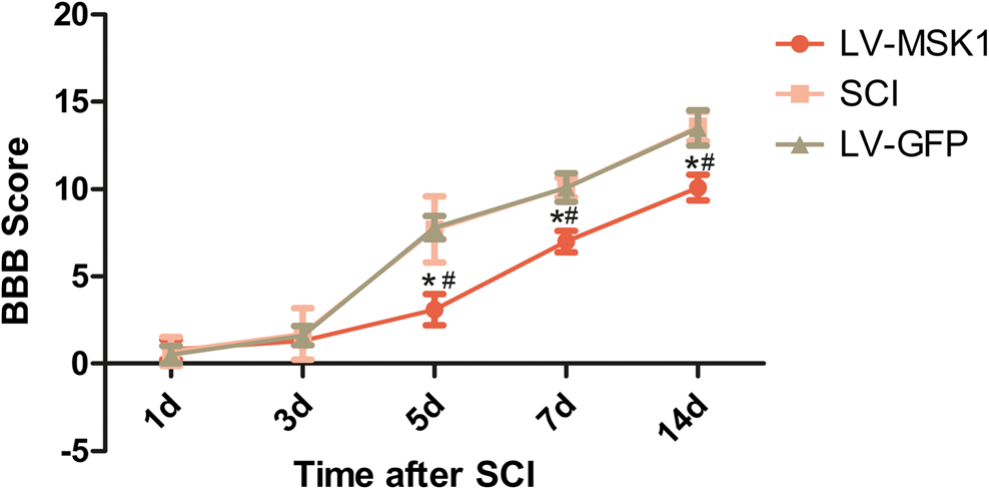
Locomotor function assessment after SCI by BBB scoring. Comparison of BBB score at 1, 3, 5, 7, and 14 days post-injury in indifferent groups. The data are mean ± SD (*n* = 5, *P < 0.05 vs. SCI group, ^#^*P* < 0.05 vs. LV-GFP group)

To determine the effect of MSK1 inhibition on the recovery of hindlimb motor function in rats, we evaluated by BBB scores. This analysis is shown in Fig. 8. We found that the BBB scores in the LV-MSK1 group were significantly lower than those in the SCI and LV-GFP groups at 5, 7, and 14 days after injury (*P* < 0.05). However, compared with those in the SCI group, BBB scores in the LV-GFP group were not significantly different at all the time points (*P* > 0.05). This indicates that inhibition of MSK1 reduces hindlimb motor functional recovery after SCI.

## Discussion

In this study, we demonstrate that MSK1 was gradually decreased after SCI at 1 day post-injury, to its lowest level at 3 days post-injury. However, after this, it gradually increased at 5 and 7 days post-injury, to reach normal levels at 14 days after injury (Fig. 1). To further investigate the effect of MSK1 in SCI and its possible mechanism, we constructed an MSK1 siRNA recombinant lentivirus. This lentiviral vector is a viral vector that has been derived from human immunodeficiency virus-1 (HIV-1), which can efficiently integrate foreign genes or exogenous shRNA into the host chromosome, thereby achieving persistent expression of the desired sequence (Olgun et al. 2018). Importantly, lentiviral vectors exhibit high neurotropism, which is a requirement for their application in the spinal cord (Tan et al. 2017).

To verify the interference efficiency of MSK1 siRNA recombinant lentivirus, well-differentiated PC12 cells were transduced with lentiviruses expressing MSK1 siRNA or LV-GFP (only expression of green fluorescent protein). The differentiated PC12 cells exhibit preserved dopaminergic characteristics and spindle-shaped cell morphology similar to neuronal cells (Liu et al. 2016; Pan et al. 2017; Chen et al. 2019). Moreover, well-differentiated PC12 cells are widely used in vitro as a model to investigate neuronal damage resulting from various hypoxia-related neurodegenerative disorders and spinal cord injury (Lin et al. 2016; Liu et al. 2016; Pan et al. 2017; Goldshmit et al. 2018; Xu et al. 2018). Well-differentiated PC12 cells were transduced with lentivirus and 72 h later, a large amount of green fluorescence was observed in the MSK1-siRNA1, MSK1-siRNA2, MSK1-siRNA3, and LV-GFP groups by fluorescence microscopy (Fig. 2). This indicates that the constructed recombinant lentiviral vectors can successfully infect well-differentiated PC12 cells. Ninety-six hours after well-differentiated PC12 cells were infected with lentivirus, the expression of MSK1 protein in the MSK1-siRNA1, MSK1-siRNA2, and MSK1-siRNA3 groups was significantly lower than that in the NC and LV-GFP groups (all *P* < 0.05; Fig. 3). This indicated that the constructed MSK1-siRNA lentiviral vector could successfully inhibit the expression of MSK1 in PC12 cells. It is worth noting that the MSK1-siRNA2 lentivirus vector had the highest interference efficiency compared with expression in the LV-GFP group. Therefore, the MSK1-siRNA2 lentivirus vector was selected for follow-up studies.

Then, we injected the MSK1-siRNA2 lentivirus locally into the spinal cords of rats. After 7 days post-injury, spontaneous green fluorescence was observed in both LV-GFP and LV-MSK1 groups (Fig. 4a). Moreover, immunofluorescence double staining showed that MSK1 colocalized with GFP and NeuN (Fig. 4a, b). In our previous study, we found that MSK1 is colocalized with GFAP (Zhong, et al. 2018). Previous studies have also reported that MSK1 is colocalized with NeuN and GFAP in brain and spinal cord tissue (Li et al. 2013; Ning et al. 2013). NeuN is a marker of neurons, and GFAP is a marker of astrocytes. Therefore, we believe that MSK1 was expressed in neurons and astrocytes. The expression of MSK1 at 7 and 14 days post-injury in the LV-MSK1 group was significantly lower than that in the SCI and LV-GFP groups by western blotting analysis (Fig. 4d, e). These results showed that the MSK1 siRNA lentivirus could effectively inhibit the expression of MSK1 in vitro and in vivo.

The inflammatory response is a critical molecular defense mechanism of the innate immune system. Recent research shows that neuroinflammation is a prominent feature of many neurodegenerative diseases and is increasingly recognized as an important pathophysiological mechanism of chronic neurodegeneration following SCI (Faden et al. 2016). In SCI, the immune barrier is impaired and immune cells invade, causing a series of inflammatory reactions such as endothelial damage, vascular permeability changes, edema, and inflammatory mediator release (Wang et al. 2016). Following the accumulation of inflammatory mediators at the site of injury, necrosis, apoptosis, and inflammation occur rapidly (Ottenweller et al. 2000). Certain inflammatory cytokines, such as TNF-α, IL-6, IL-10, and IL-1β are important mediators of spinal cord inflammation (Keane et al. 2006). This involves multiple signaling pathways, which induce a cascade that amplifies the inflammatory response, ultimately leading to neuronal necrosis (Qu et al. 2012; Deak et al. 1998). However, experiments have shown that blocking various inflammatory cascades can alleviate experimental SCI (Gonzalez et al. 2003; Cronin et al. 2008; Tian et al. 2009). During immune processes, MSK1 has predominantly anti-inflammatory roles and helps to regulate the production of the anti-inflammatory cytokine IL-10 (Reyskens and Arthur 2016). In macrophages, MSK1 is involved in negative feedback pathways. It can affect the secretion of TNF-α/IL-6/IL-1β by regulating IL-10, which is critical to prevent uncontrolled inflammation. (Ananieva et al. 2008). In a brain injury model induced by bacterial lipopolysaccharide in rats, MSK1 was also found to be involved in astrocyte inflammation and inhibit the production of inflammatory cytokines TNF-α, IL-6, and IL-1β (Gong et al. 2013). Whether MSK1 is involved in the inflammatory response after spinal cord injury and if it regulates the secretion of inflammatory cytokines was not known. Therefore, in this study, we determined the expression of proinflammatory cytokines TNF-α, IL-6, and IL-1β and the anti-inflammatory cytokine IL-10 in different groups by western blotting. We found that after inhibiting the expression of MSK1 in the spinal cord, the expression levels of TNF-α, IL-6, and IL-1β were significantly increased (Fig. 5a–c) and the expression of IL-10 was decreased (Fig. 5e). The balance between the inflammatory response and its resolution is maintained in part by the activity of two transcription factors with opposite roles, namely NF-κB and CREB (Huante-Mendoza et al. 2016). Activation of NF-κB induces the expression of pro-inflammatory markers (e.g., TNF-α, IL-6, IL-8, and COX-2), whereas the activation of CREB leads to the expression of cytokines that have an anti-inflammatory role (e.g., IL-4, IL-10, and IL-13). CREB and NF-κB p65 are phosphorylated by MSK1, thereby enhancing their transcriptional activity. Therefore, we believe that MSK1 might participate in the inflammatory response during spinal cord injury and regulate the production of inflammatory cytokines by phosphorylating NF-κB and CREB.

Apoptosis, also known as programmed cell death, might play a pivotal role in secondary SCI (Emery et al. 1998). It can be triggered by a variety of mechanisms including glutamate excitotoxicity, free radical damage, cytokines, and inflammatory damage (Lu et al. 2000). The apoptotic cascade is activated in neurons, oligodendrocytes, microglia, and astrocytes (Dumont et al. 2001). In these cells, apoptosis induced by caspase plays an important role in SCI (Springer et al. 1999). Studies have shown that MSK1 plays a decisive role in apoptosis (Mellidis et al. 2014; Lang et al. 2015). Further, it is associated with Mn^2+^-induced cell death in human B cells, leading to caspase-3 activity and apoptosis (El Mchichi et al. 2007).

Caspases are well recognized as crucial apoptosis regulators and are generally classified into two groups, specifically the initiator caspases (including caspase-2, caspase-8, caspase-9, and caspase-10) and executioner caspases (comprising caspase-3, caspase-6, and caspase-7) (Yakovlev and Faden 2001; Eldadah and Faden 2009). Caspase-3, an effector caspase, is activated by extrinsic and intrinsic apoptosis pathways and its activation ultimately leads to cell death. (Slee et al. 2001; Samuel et al. 2006; Hashemi et al. 2018). A previous study showed that the inhibition of p38 MAPK significantly decreases the phosphorylation of MSK1, increases cleaved caspase-3 expression, and abolishes neuroprotection upon hypoxic post-conditioning (HPC) (Zhu et al. 2014). This indicates that MSK1 might contribute to HPC-mediated neuroprotection against transient global cerebral ischemia by regulating the activation of caspase-3. MSK1 has also been suggested to regulate cell-death-associated proteins and translation initiation. Bad (B cell lymphoma 2 (Bcl-2)-associated death protein) is a member of the pro-apoptotic Bcl-2 family. Knockdown of MSK1 was found to suppress Bad phosphorylation after calcium ionophore (A23187) treatment in neuronal cells (Clark et al. 2007). Recent research has also shown that MSK1 might be involved in SAH and TBI-induced apoptosis. (Ning et al. 2013, 2017). However, whether it is related to neuronal apoptosis caused by SCI was not clear. In this study, immunofluorescence double staining showed that that the number of NeuN^+^ caspase-3-positive cells was significantly higher than that in the LV-GFP group (Fig. 6a, c). In addition, TUNEL staining (Fig. 6b, d) indicated that the number of TUNEL-positive cells in the LV-MSK1 group was evidently increased compared with that in the LV-GFP group. Based on transmission electron microscopy to observe the morphology of nerve cells, injured spinal cords in the SCI, LV-GFP, and LV-MSK1 groups, at 14 days post-injury (Fig. 7) showed characteristics of neuronal apoptosis such as condensed nuclei, concentrated chromatin, wrinkled nuclear membrane, irregularly shapes, and thin myelin, compared with that observed in normal spinal cord tissue. However, in the LV-MSK1 group, neurons exhibited severely damaged cell membranes, swollen mitochondria, and structurally disrupted myelin with demyelination. These data indicate that with MSK1 inhibition, apoptosis is increased and neurons suffer significant damage.

Moreover, we measured the movement of hind limbs in rats based on BBB scores at 1, 3, 5, 7, and 14 days after SCI. The scores at 5, 7, and 14 days post-injury in the LV-MSK1 group were lower than those in the LV-GFP and SCI groups (Fig. 8). Traumatic injury to the CNS results in a rapid inflammatory response by the resident astrocytes, characterized primarily by hypertrophy and proliferation, which results in the release of inflammatory and cytotoxic substances (Myer et al. 2006). Although inflammation is the physiological process through which vascularized tissues respond to injury, the overactivation of this process can lead to the excessive activation of astrocytes and the aggravation of neuronal necrosis (Brambilla et al. 2005; Graeber et al. 2011). In severe cases, this will eventually lead to the formation of glial scars around the injury site and the secretion of a variety of neurotoxic molecules, preventing post-injury neuronal repair and axonal regeneration and ultimately resulting in worse clinical outcome (Brambilla et al. 2005). In a previous study, we showed that the inhibition of MSK1 promotes active astrocyte proliferation (Zhong et al. 2018). MSK1 might also play a critical role in the repair of SCI in rats by regulating the proliferation of glial cells. In this study, after MSK1 inhibition, the expression of proinflammatory cytokines was significantly increased and the expression of an anti-inflammatory cytokine was decreased. In addition, neuronal apoptotic cells were increased significantly and ultrastructural analysis of nerve cells also revealed severe neuronal damage. Mechanistically, on the one hand, the inhibition of MSK1 promotes inflammatory responses, leading to the formation of glial scars around the injury site, preventing post-injury neuronal repair and axonal regeneration. On the other hand, the inhibition of MSK1 promotes neuronal apoptosis, resulting in a decrease in the number of spinal motor neurons at the site of injury. As a result, hind limb movement in rats was obviously worse.

In conclusion, the results of this study indicate that the constructed MSK1 siRNA recombinant lentivirus can be successfully transduced into rat spinal cords and effectively inhibit the expression of MSK1. More importantly, inhibition of MSK1 promotes inflammatory responses and apoptosis and reduces functional recovery after SCI in rats. Therefore, our findings provide evidence that MSK1 might play an important role in the repair of SCI by regulating inflammation and apoptosis.

## Electronic supplementary material

ESM 1 (DOCX 2581 kb)

## References

[CR1] Allen AR (1911). Surgery of experimental lesion of spinal cord equivalent to crush injury of fracture dislocation of spinal column: a preliminary report. J Am Med Assoc.

[CR2] Ananieva O, Darragh J, Johansen C, Carr JM, McIlrath J, Park JM, Wingate A, Monk CE, Toth R, Santos SG, Iversen L, Arthur JSC (2008). The kinases MSK1 and MSK2 act as negative regulators of toll-like receptor signaling. Nat Immunol.

[CR3] Arthur JSC (2008). MSK activation and physiological roles. Front Biosci.

[CR4] Basso DM, Beattie MS, Bresnahan JC (2009). A sensitive and reliable locomotor rating scale for open field testing in rats. J Neurotrauma.

[CR5] Brambilla R, Bracchi-Ricard V, Hu W-H, Frydel B, Bramwell A, Karmally S, Green EJ, Bethea JR (2005). Inhibition of astroglial nuclear factor κB reduces inflammation and improves functional recovery after spinal cord injury. J Exp Med.

[CR6] Chen X-H, Chen D-T, Huang X-M, Chen YH, Pan JH, Zheng XC, Zeng WA (2019). Dexmedetomidine protects against chemical hypoxia-induced neurotoxicity in differentiated PC12 cells via inhibition of NADPH oxidase 2-mediated oxidative stress. Neurotox Res.

[CR7] Cheng P, Alberts I, Li X (2013). The role of Erk1/2 in the regulation of proliferation and differentiation of astrocytes in developing brain. Int J Dev Neurosci.

[CR8] Clark CJ, McDade DM, O’Shaughnessy CT, Morris BJ (2007). Contrasting roles of neuronal Msk1 and Rsk2 in Bad phosphorylation and feedback regulation of Erk signalling. J Neurochem.

[CR9] Cronin M, Anderson PN, Cook JE, Green CR, Becker DL (2008). Blocking connexin43 expression reduces inflammation and improves functional recovery after spinal cord injury. Mol Cell Neurosci.

[CR10] Deak M, Clifton AD, Lucocq JM, Alessi DR (1998). Mitogen- and stress-activated protein kinase-1 (MSK1) is directly activated by MAPK and SAPK2/p38, and may mediate activation of CREB. EMBO J.

[CR11] Dumont RJ, Okonkwo DO, Verma S, Hurlbert RJ, Boulos PT, Ellegala DB, Dumont AS (2001). Acute spinal cord injury, part I: pathophysiologic mechanisms. Clin Neuropharmacol.

[CR12] El Mchichi B, Hadji A, Vazquez A, Leca G (2007). p38 MAPK and MSK1 mediate caspase-8 activation in manganese-induced mitochondria-dependent cell death. Cell Death Differ.

[CR13] Eldadah BA, Faden AI (2009). Caspase pathways, neuronal apoptosis, and CNS injury. J Neurotrauma.

[CR14] Emery E, Aldana P, Bunge MB, Puckett W, Srinivasan A, Keane RW, Bethea J, Levi ADO (1998). Apoptosis after traumatic human spinal cord injury. J Neurosurg.

[CR15] Faden AI, Wu J, Stoica BA, Loane DJ (2016). Progressive inflammatory-mediated neurodegeneration after traumatic brain or spinal cord injury. Br J Pharmacol.

[CR16] Goldshmit Y, Tang JKKY, Siegel AL, Nguyen PD, Kaslin J, Currie PD, Jusuf PR (2018). Different Fgfs have distinct roles in regulating neurogenesis after spinal cord injury in zebrafish. Neural Dev.

[CR17] Gong P, Xu X, Shi J, Ni L, Huang Q, Xia L, Nie D, Lu X, Chen J, Shi W (2013). Phosphorylation of mitogen- and stress-activated protein kinase-1 in astrocytic inflammation: a possible role in inhibiting production of inflammatory cytokines. PLoS One.

[CR18] Gonzalez R, Glaser J, Liu MT, Lane TE, Keirstead HS (2003). Reducing inflammation decreases secondary degeneration and functional deficit after spinal cord injury. Exp Neurol.

[CR19] Graeber MB, Li W, Rodriguez ML (2011). Role of microglia in CNS inflammation. FEBS Lett.

[CR20] Hashemi M, Moazeni-Roodi A, Ghavami S (2018) Association between CASP3 polymorphisms and overall cancer risk: a meta-analysis of case-control studies. J Cell Biochem. 10.1002/jcb.27994 10.1002/jcb.27994 30368918

[CR21] Hu J, Lang Y, Zhang T, Ni S, Lu H (2016). Lentivirus-mediated PGC-1α overexpression protects against traumatic spinal cord injury in rats. Neuroscience..

[CR22] Huante-Mendoza A, Silva-García O, Oviedo-Boyso J, Hancock REW, Baizabal-Aguirre VM (2016). Peptide IDR-1002 inhibits NF-κB nuclear translocation by inhibition of IκBα degradation and activates p38/ERK1/2-MSK1-dependent CREB phosphorylation in macrophages stimulated with lipopolysaccharide. Front Immunol.

[CR23] Kaiser M, Wiggin GR, Lightfoot K (2007). MSK regulate TCR-induced CREB phosphorylation but not immediate early gene transcription. Eur J Immunol.

[CR24] Keane RW, Davis AR, Dietrich WD (2006). Inflammatory and apoptotic signaling after spinal cord injury. J Neurotrauma.

[CR25] Kim HG, Ki WL, Cho YY (2008). Mitogen- and stress-activated kinase 1-mediated histone H3 phosphorylation is crucial for cell transformation. Cancer Res doi.

[CR26] Lang E, Bissinger R, Fajol A, Salker MS, Singh Y, Zelenak C, Ghashghaeinia M, Gu S, Jilani K, Lupescu A, Reyskens KMSE, Ackermann TF, Föller M, Schleicher E, Sheffield WP, Arthur JSC, Lang F, Qadri SM (2015) Accelerated apoptotic death and in vivo turnover of erythrocytes in mice lacking functional mitogen- and stress-activated kinase MSK1/2. Sci Rep. 10.1038/srep17316 10.1038/srep17316 PMC4661433 26611568

[CR27] Li Z, Zhao L, Hang H, Zhu N, Ning B, Lv Z (2013). Spatiotemporal patterns and essential role of MSK1 expression after rat spinal cord injury. Neurochem Res.

[CR28] Lin C-W, Chen B, Huang K-L, Dai YS, Teng HL (2016). Inhibition of autophagy by estradiol promotes locomotor recovery after spinal cord injury in rats. Neurosci Bull.

[CR29] Liu X, Zhu X, Chen M, Ge Q, Shen Y, Pan S (2016). Resveratrol protects PC12 cells against OGD/R-induced apoptosis via the mitochondrial-mediated signaling pathway. Acta Biochim Biophys Sin Shanghai.

[CR30] Lu J, Ashwell KWS, Waite P (2000). Advances in secondary spinal cord injury: role of apoptosis. Spine (Phila. Pa. 1976).

[CR31] Mellidis K, Dokalis N, Barlaka E, Lazou A (2014). P87Activation of mitogen and stress activated kinase 1 (MSK1) during oxidative stress modulates apoptotic and autophagy pathways leading to cardioprotection. Cardiovasc Res.

[CR32] Myer DJ, Gurkoff GG, Lee SM (2006). Essential protective roles of reactive astrocytes in traumatic brain injury. Brain..

[CR33] Ning B, Li Z, Zhu N, Hou G, Pang Q (2013). Traumatic brain injury induces a downregulation of MSK1 in rat brain cortex. J Mol Neurosci.

[CR34] Ning B, Guo G, Liu H, Ning L, Sun BL, Li Z, Wang S, Lv ZW, Fan CD (2017). MSK1 downregulation is associated with neuronal and astrocytic apoptosis following subarachnoid hemorrhage in rats. Oncol Lett.

[CR35] Olgun HB, Tasyurek HM, Sanlioglu AD, Sanlioglu S (2018). High-titer production of HIV-based lentiviral vectors in roller bottles for gene and Cell therapy.

[CR36] Ottenweller JE, Li M-T, Giglio W, Anesetti R, Pogach LM, Huang HFS (2000). Alteration of follicle-stimulating hormone and testosterone regulation of messenger ribonucleic acid for Sertoli cell proteins in the rat during the acute phase of spinal cord Injury1. Biol Reprod.

[CR37] Pan X, Yan D, Wang D, Wu X, Zhao W, Lu Q, Yan H (2017). Mitochondrion-mediated apoptosis induced by acrylamide is regulated by a balance between Nrf2 antioxidant and MAPK signaling pathways in PC12 cells. Mol Neurobiol.

[CR38] Qu W, Tian D, Guo Z, Fang J, Zhang Q, Yu ZY, Xie MJ, Zhang HQ, Lü JG, Wang W (2012). Inhibition of EGFR/MAPK signaling reduces microglial inflammatory response and the associated secondary damage in rats after spinal cord injury. J Neuroinflammation.

[CR39] Reyskens KMSE, Arthur JSC (2016) Emerging roles of the mitogen and stress activated kinases MSK1 and MSK2. Front Cell Dev Biol. 10.3389/fcell.2016.00056 10.3389/fcell.2016.00056 PMC4901046 27376065

[CR40] Rouanet C, Reges D, Rocha E, Gagliardi V, Silva GS (2017). Traumatic spinal cord injury: current concepts and treatment update. Arq Neuropsiquiatr.

[CR41] Saghazadeh A, Rezaei N (2017). The role of timing in the treatment of spinal cord injury. Biomed Pharmacother.

[CR42] Samuel MA, Morrey JD, Diamond MS (2006). Caspase 3-dependent cell death of neurons contributes to the pathogenesis of West Nile virus encephalitis. J Virol.

[CR43] Simon J (2004). Mitogen- and stress-activated protein kinase 1 mediates cAMP response element-binding protein phosphorylation and activation by neurotrophins. J Neurosci.

[CR44] Slee EA, Adrain C, Martin SJ (2001). Executioner caspase-3, -6, and -7 perform distinct, non-redundant roles during the demolition phase of apoptosis. J Biol Chem.

[CR45] Springer JE, Azbill RD, Knapp PE (1999). Activation of the caspase-3 apoptotic cascade in traumatic spinal cord injury. Nat Med.

[CR46] Tan BT, Jiang L, Liu L, Yin Y, Luo ZRX, Long ZY, Li S, Yu LH, Wu YM, Liu Y (2017). Local injection of Lenti-Olig2 at lesion site promotes functional recovery of spinal cord injury in rats. CNS Neurosci Ther.

[CR47] Tian DS, Liu JL, Xie MJ, Zhan Y, Qu WS, Yu ZY, Tang ZP, Pan DJ, Wang W (2009). Tamoxifen attenuates inflammatory-mediated damage and improves functional outcome after spinal cord injury in rats. J Neurochem.

[CR48] Ueno M, Yamashita T (2008). Strategies for regenerating injured axons after spinal cord injury - insights from brain development. Biologics.

[CR49] Vermeulen L, De Wilde G, Van Damme P (2003). Transcriptional activation of the NF-κB p65 subunit by mitogen- and stress-activated protein kinase-1 (MSK1). EMBO J.

[CR50] Vermeulen L, Vanden Berghe W, Beck IME (2009). The versatile role of MSKs in transcriptional regulation. Trends Biochem Sci.

[CR51] Wang J, Su B, Zhu H, Chen C, Zhao G (2016). Protective effect of geraniol inhibits inflammatory response, oxidative stress and apoptosis in traumatic injury of the spinal cord through modulation of NF-κb and p38 MAPK. Exp Ther Med.

[CR52] Wang H, Liu X, Li R, Zhang P, Chu Z, Wang CL, Liu HR, Qi J, Lv GY, Wang GY, Liu B, Li Y, Wang YY (2017). Effect of glial cells on remyelination after spinal cord injury. Neural Regen Res.

[CR53] Wiggin GR, Soloaga A, Foster JM, Murray-Tait V, Cohen P, Arthur JSC (2002). MSK1 and MSK2 are required for the mitogen- and stress-induced phosphorylation of CREB and ATF1 in fibroblasts. Mol Cell Biol.

[CR54] Xu Y, Kabba JA, Ruan W, Wang Y, Zhao S, Song X, Zhang L, Li J, Pang T (2018). The PGC-1α activator ZLN005 ameliorates ischemia-induced neuronal injury in vitro and in vivo. Cell Mol Neurobiol.

[CR55] Yakovlev AG, Faden AI (2001). Caspase-dependent apoptotic pathways in CNS injury. Mol Neurobiol.

[CR56] Zhong Z, Zhou Y, Feng S, Huang Y, Chen X (2018). [Effect of lentivirus-mediated small interfering RNA on mitogen- and stress-activated protein kinase 1 in spinal cord injury of rats]. Zhongguo Xiu Fu Chong Jian Wai Ke Za Zhi.

[CR57] Zhu P, Zhan L, Zhu T, Liang D, Hu J, Sun W, Hou Q, Zhou H, Wu B, Wang Y, Xu E (2014). The roles of p38 MAPK/MSK1 signaling pathway in the neuroprotection of hypoxic postconditioning against transient global cerebral ischemia in adult rats. Mol Neurobiol.

