# Supplementary material for: Inhibition of MSK1 Promotes Inflammation and Apoptosis and Inhibits Functional Recovery After Spinal Cord Injury
Source: J Mol Neurosci. 2019 Mar 27;68(2):191–203. doi: 10.1007/s12031-019-01298-9 (PMC6511344; doi:10.1007/s12031-019-01298-9)
Supplement: Supplementary file 1 — (DOCX 2581 kb) [file 12031_2019_1298_MOESM1_ESM.docx]

**(1) Construction of LV3-shRNA**

Design DNA oligo using Designer3.0 software. The loop structure in the LV3-shRNA template uses TTCAAGAGA to avoid the formation of a termination signal. GATCC was added to the 5' end of the positive-sense strand template, which is complementary to the sticky end formed by BamHI digestion. AATTC was added to the 5' end of the antisense strand template, which is complementary to the sticky end formed by EcoRI digestion.

Positive-sense strand：5’-GATCC-(GN18) -(TTCAAGAGA) -(N18C)-TTTTTTG- 3’

Antisense strand: 3’-G(CN18) -(AAGTTCTCT) -(N18G)-AAAAAACTTAA-5’

The three shRNA sequences targeting the rat MSK1 sequence (GenBank NM_001108048.1) were designed as follows:

KD1:5′-GCGTTTCACAGAGCACGAAGT-3′

KD2: 5′- GGAATGAGCTCAGTAGCTAAA-3′

KD3: 5′- GCTCCTTCCATCCTCTTCAAG-3′

**(2) Annealing of LV3-shDNA template**

The DNA oligo was dissolved in TE (pH 8.0) at a concentration of 100 μM.


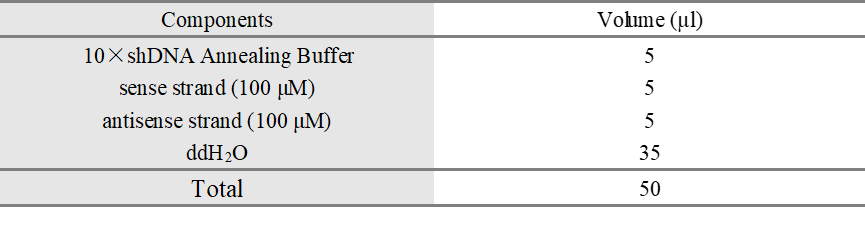
The corresponding positive-sense chain and antisense strand oligo solution were taken, and the annealing reaction system was configured according to the following ratio:

Annealing on the PCR machine according to the following procedure: 95℃ 5min; 85℃ 5min; 75℃ 5min; 70℃ 5min; Store at 4 ° C. After annealing, a shRNA template with a concentration of 10 μM was obtained. The resulting template solution was diluted 50 times to a final concentration of 200 nM for ligation reactions.

**(3) Linearization of LV3 carrier**

Take 10 μg of LV3 vector and perform enzyme digestion according to the following system:

Enzymatic digestion at 37 ° C for 1 hour, agarose electrophoresis, Recycling with Agarose Gel DNA Purification Kit Ver2.0, Electrophoretic detection estimated concentration, diluted to 50
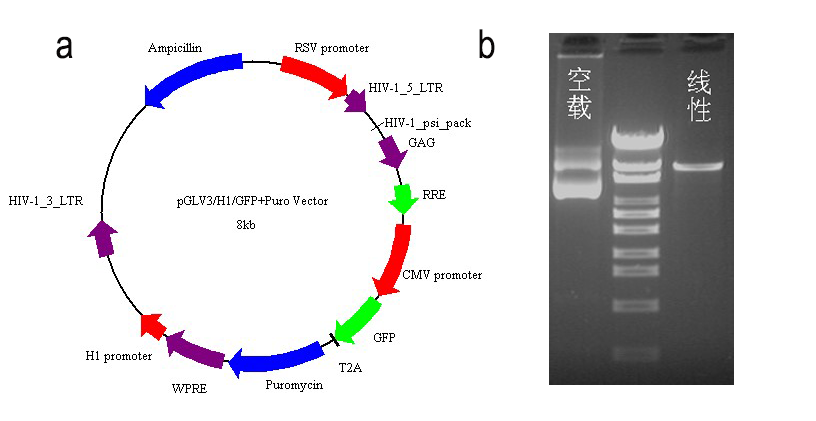
ng / μl.

| Components | Volume (µl) |
| --- | --- |
| 10×Buffer Tango | 10 |
| BamHI | 5 |
| EcoRI | 5 |
| LV3 vector | 10 µg |
| ddH_2_O | To 100 µl |
| Total | 100 |

Fig1: a Structure of LV3 vector; b Agarose electrophoresis picture

**(4) Construction of LV3-shRNA vector**


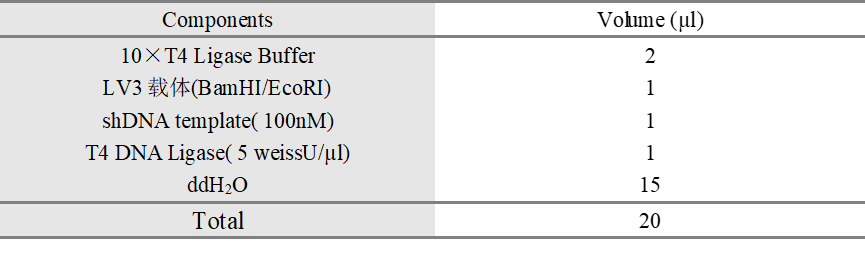
Carrying out the ligation reaction of the carrier according to the following system:

**(5) Preparation of competent cells by calcium chloride method** (Reference Molecular Cloning, A Guide to Experiments, Second Edition, 55 pages)

**(6) Transformation of the ligation product**

I: Retrieve competent cells from -70 ° C, after the cells are thawed, add 10 μl of ligation product, and mix the content

II: Add 800μl LB medium (without antibiotics). Then transfer the tube to a 37 ° C shaker, 250 rpm, incubated for 45 minutes to resuscitate the bacteria

III: 200 μl of the cultured cells were uniformly coated on a 50 μg/ml Ampicillin LB plate.

IV: After the liquid on the plate is absorbed, the plate is placed in a 37 ° C incubator and cultured for 16 hours.

**(7) Identification and sequencing of positive clones**

I: Four colonies were picked from each plate, inoculated into LB medium containing 50 μg/ml Ampicillin, and cultured at 37 ° C for 16 hours.

II: Extraction of the plasmid using alkaline lysis


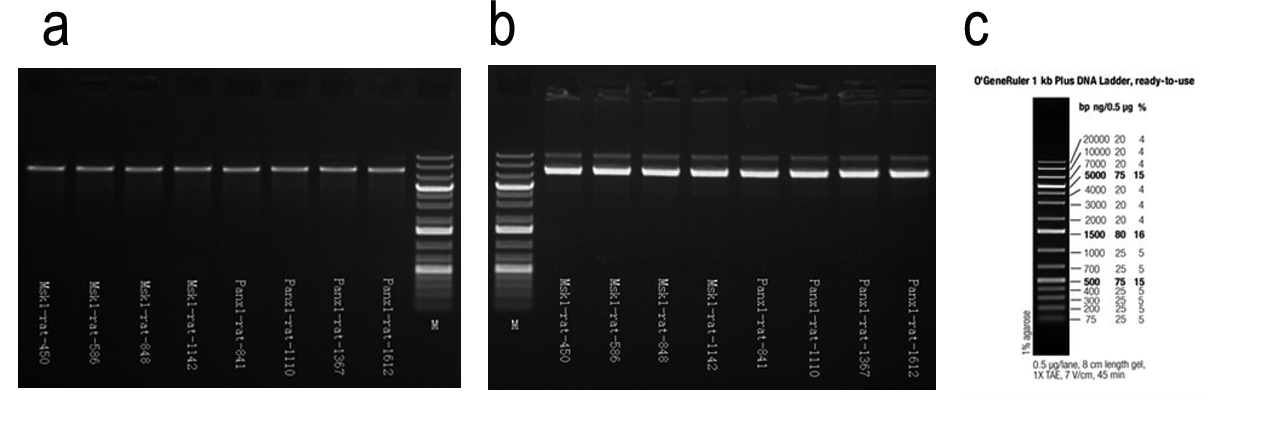
III: The obtained plasmid was identified by single enzyme digestion with EcoRI

Fig2: The results of the digestion showed that the positive clones were cut by EcoRI.

a Restriction map of recombinant vector; b Recombinant vector plasmid map; c DNA Marker. M: 1Kb

**(8)** **Sequencing and identification of clones. (figure3-5)**

**(9) Collection of lentiviral supernatants**

I: Sequencing the correct strain using a high-purity plasmid medium extraction kit for extraction

II: The resulting plasmid was mixed with the packaging plasmid (pGag/Pol, pRev, Pvsv-G) and RNAi-mate. Co-transfection of 293Tcell, followed by collection of lentiviral supernatants. Store at -80 ° C after dispensing.

**(10) Determination of lentivirus titer**

Lentiviral supernatant transfected with 293T cells at different dilutions; Calculation of fluorescent cells by fluorescence microscopy; Calculate lentivirus titer by combining the dilution factor of lentivirus. The three constructed lentiviral titers were 0.9 × 10^9^ TU/mL.(Fig3-
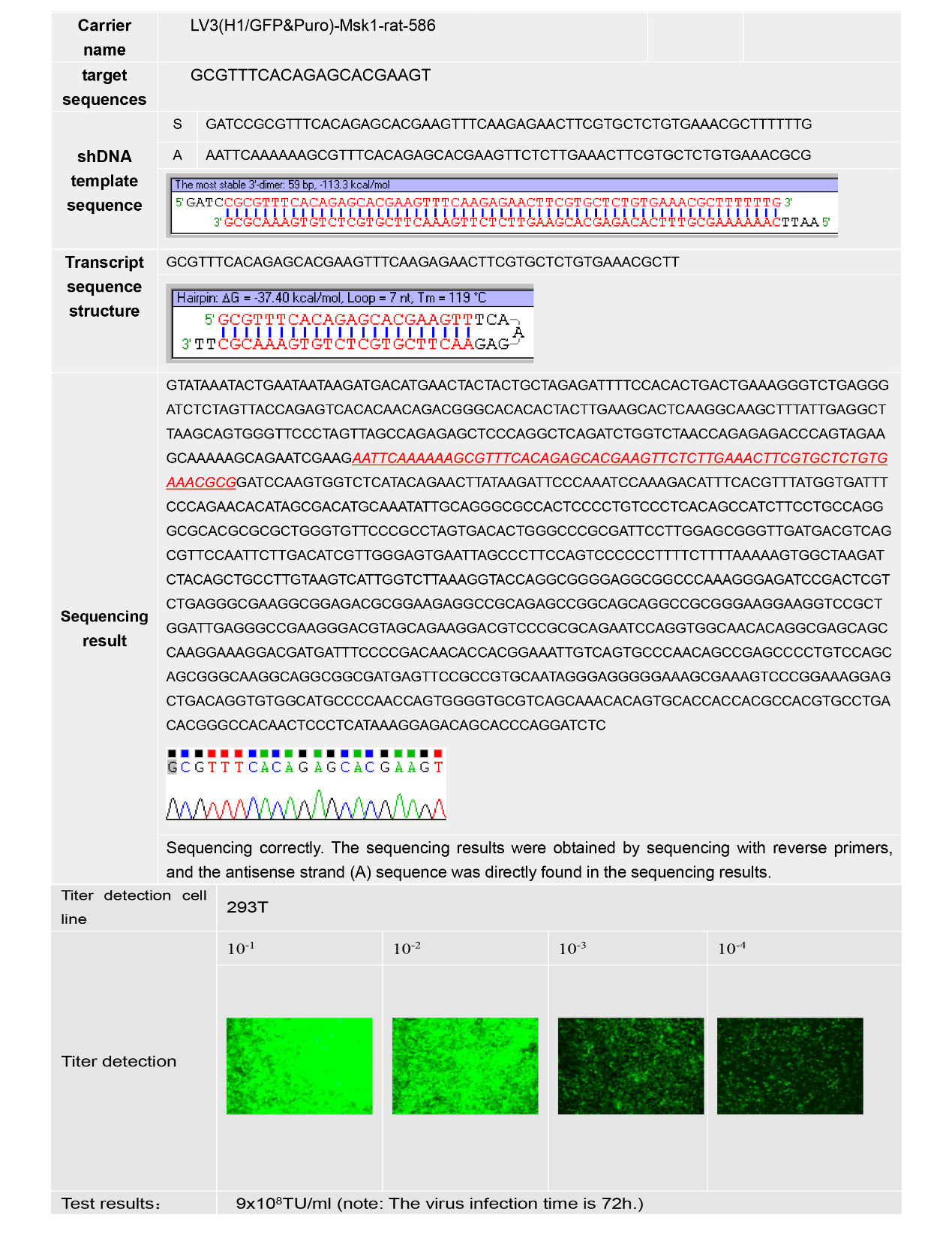
5)

Fig3: Sequencing result and titer detection of KD1


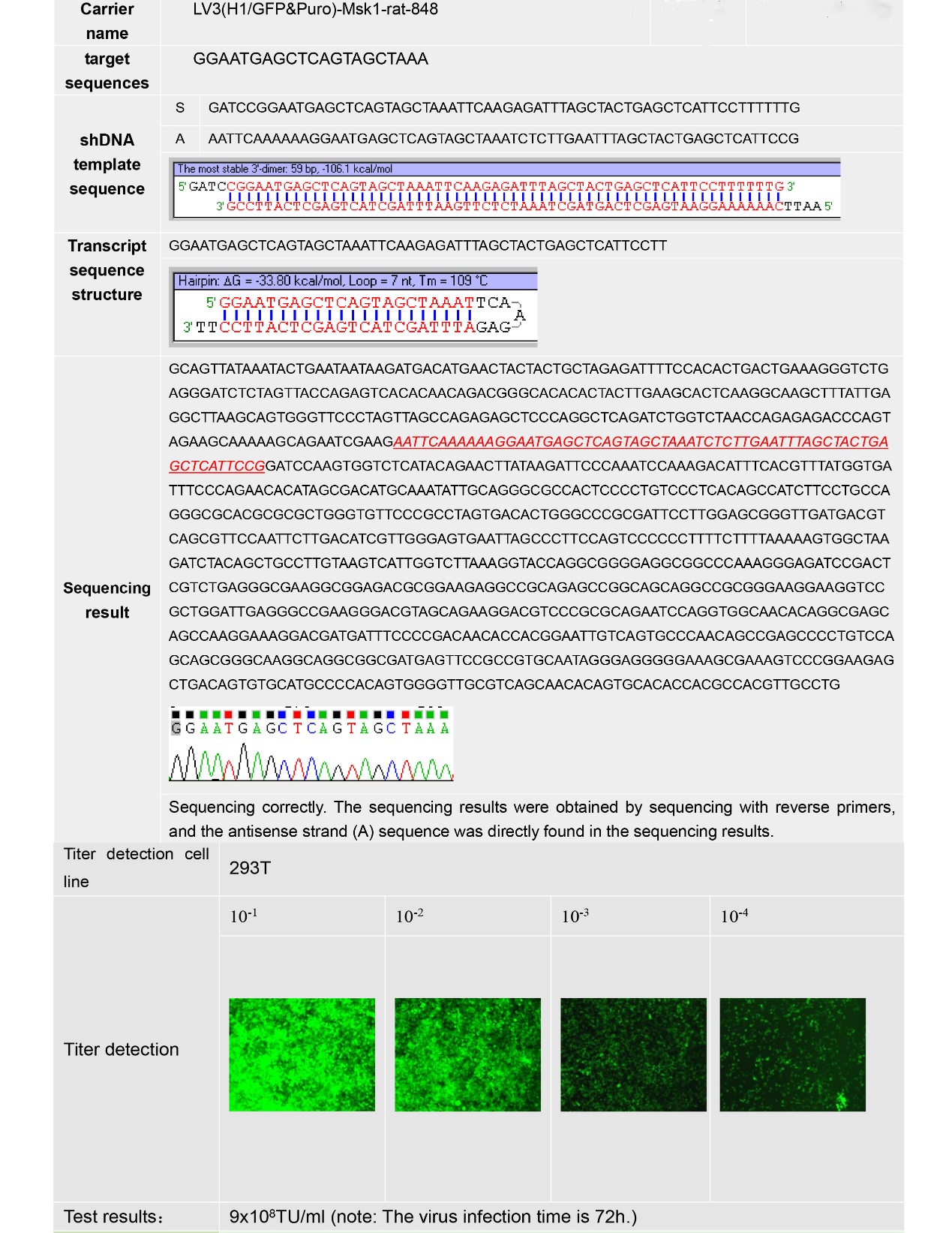
 Fig4: Sequencing result and titer detection of KD2


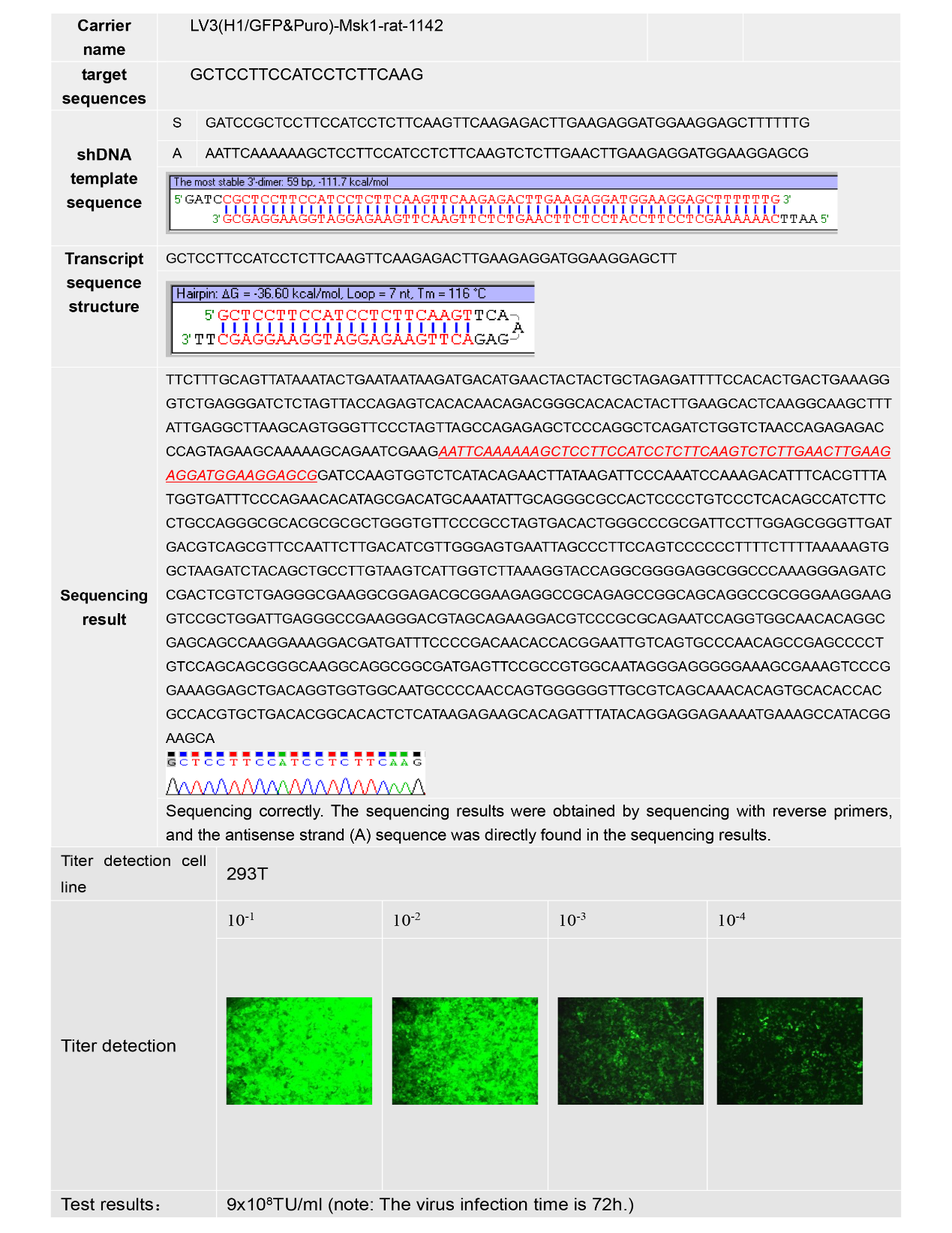
 Fig4: Sequencing result and titer detection of KD3
